# Effects of Extrusion on the Available Energy and Nutrient Digestibility of Wheat and Its Application in Weaned Piglets

**DOI:** 10.3390/ani15040528

**Published:** 2025-02-12

**Authors:** Ge Zhang, Xing He, Zhiqiang Sun, Tenghao Wang, Ling Liu, Jinbiao Zhao, Zeyu Zhang

**Affiliations:** 1 State Key Laboratory of Animal Nutrition and Feeding, College of Animal Science and Technology, China Agricultural University, Beijing 100193, China; zhangge0557@163.com (G.Z.); sunbeibei5421@163.com (Z.S.); jinbiaozhao@cau.edu.cn (J.Z.); 2 Zhejiang Qinglian Food Co., Ltd., Jiaxing 314399, China

**Keywords:** wheat, extrusion, weaned piglets, available energy, growth performance, fecal microbiota

## Abstract

Experiments were designed to investigate the effect of extrusion on the available energy and nutrient digestibility of wheat, and to further evaluate the effects of incorporating extruded wheat into the diet on growth performance, diarrhea rates, health status, and fecal microbiota of weaned piglets. In Exp. 1, twelve crossbred barrows, with an initial body weight (BW) of 12.0 ± 0.73 kg, were randomly assigned to two treatment groups (wheat and extruded wheat) to determine the digestible energy (DE) and metabolizable energy (ME). Additionally, nine crossbred barrows (initial BW, 13.73 ± 0.59 kg) fitted with ileal cannulas, were arranged in a 9 × 2 Youden square design, including three diets (N-free diet, wheat, and extruded wheat), aiming to measure the standard ileal digestibility of amino acids (AAs). In Exp. 2, 96 pigs (initial BW, 8.58 ± 0.52 kg) were randomly divided into two treatments (CON and 35% extruded wheat) based on BW, with each treatment having six pens, each pen containing four boars and four sows. The results indicated that extrusion significantly increased the DE and ME of wheat and the standard ileal digestibility of most AAs (*p* < 0.05). Incorporating 35% extruded wheat did not affect the growth performance, diarrhea rates, nutrient digestibility, or health status of the weaned piglets (*p* > 0.05). However, it tended to enhance the α-diversity of fecal microbiota (*p* = 0.07) and increased the relative abundance of beneficial bacteria (*p* < 0.05). In conclusion, extrusion increased the feeding value of wheat, and the inclusion of 35% extruded wheat in the diet increased the relative abundance of beneficial microbes in feces.

## Simple Summary

Wheat holds considerable potential as a substitute for corn; however, its high content of non-starch polysaccharides presents a significant challenge when incorporated heavily into the diets of weaned piglets. This study, approached from the perspective of processing, evaluates the effects of extrusion on the digestible energy, metabolizable energy, and digestibility of amino acids in wheat. Based on these results, further investigation was conducted on the effects of inclusion of 35% extruded wheat to the diet on growth performance, diarrhea rates, nutrient digestibility, health status, and fecal microbiota of weaned piglets.

## 1. Introduction

In recent years, the rapid development of swine farming has exacerbated the shortage of corn resources and led to rising prices, posing significant challenges for the livestock industry [1]. Consequently, there has been increasing research focused on identifying alternative feed ingredients to replace corn. Wheat has emerged as a promising candidate, as it can provide pigs with a substantial portion of indispensable amino acids, fulfilling up to 60% of the total amino acid (AA) requirements and as much as 70% of indispensable AAs [2]. In Europe, approximately 33% of wheat production is utilized as animal feed. Additionally, wheat processing by-products, such as dried wheat distillers’ grains with solubles and wheat bran, are extensively incorporated into livestock and poultry feeds [3]. However, it is important to note that wheat contains a high level of non-starch polysaccharides (NSPs) such as arabinoxylans, β-glucans, and pectic polysaccharides [4]. The absence of endogenous enzymes capable of hydrolyzing NSPs in pigs often results in the encapsulation of other nutrients, thereby reducing their digestibility and potentially leading to an increase in deleterious microorganisms in the large intestine [5,6]. Therefore, supplementation with exogenous enzymes is often necessary to enhance the digestibility and utilization of nutrients in feeds containing NSPs [7,8,9,10].

Over recent years, considerable attention has been given to improving the nutritional value of feed ingredients through various processing techniques. These methods notably enhanced the digestibility of nutrients and energy, leading to improved swine growth performance [11]. Extrusion stands out as an innovative technology that not only boosts the nutritional value of feed ingredients but also fortifies their stability [12,13]. Notably, the extrusion process influences a broad spectrum of nutrients, encompassing starch, NSPs, protein, and fats [14,15,16,17].

We hypothesize that extrusion can improve AA digestibility, increase available energy, and degrade NSPs in wheat. This study aimed to investigate the effects of extrusion on the digestible energy (DE), metabolizable energy (ME), and standardized ileal digestibility (SID) of AAs in wheat. Furthermore, the experiment explored the impact of incorporating extruded wheat into the diet of weaned piglets, evaluating growth performance, incidence of diarrhea, nutrient digestibility, and fecal microbiota composition.

## 2. Materials and Methods

### 2.1. Ethics Statements

The experimental protocol was approved by the Institutional Animal Care and Use Committee of China Agricultural University (Beijing, China). The protocol was based on the National Research Council’s Guide for the Care and Use of Laboratory Animals (CAU AW82212202-1-4).

### 2.2. Wheat and Device Selection

In the experiments, the wheat was supplied by Henan Shennong Feed Technology Co., Ltd. (Zhengzhou, China). The extrusion device, as described in a previous study [18], was a twin-screw extruder purchased from Beijing Modern Yang Industrial Mechanical Technology Development Co., Ltd. (TPE62S, Beijing, China), featuring a barrel diameter of 200 mm and an aspect ratio (L/D) of 20. This extruder equipment was used with slightly modified process parameters: the main shaft speed was set at 550 r/min, the feeding frequency was 15 Hz, and the temperature at the extruder section was maintained at 140 °C. After cooling, the material was crushed and sieved through a 1.0 mm screen.

### 2.3. Animals, Diets and Experimental Designs

Exp. 1 aimed to evaluate the effects of extrusion on the digestible energy (DE), metabolizable energy (ME), and amino acid (AA) digestibility of wheat. Twelve crossbred barrows (Duroc × (Landrace × Large White)) at 40 days of age, with an initial body weight (BW) of 12.0 ± 0.73 kg, were allocated into two groups (wheat and extruded wheat) based on BW, with six pigs in each group. The experiment included a 7 d adaptation period followed by a 5 d sample collection period. Dietary compositions and nutritional contents for the digestion metabolism experiment are provided in Table 1. Following the methodology adapted from Stein et al. [19], cannulas were surgically installed in the distal ileum of nine crossbred barrows, which had an initial BW of 13.73 ± 0.59 kg. After a 14 d recovery period, the pigs were divided into three groups, each consisting of three pigs, based on BW. A 9 × 2 Youden square design was employed, involving nine pigs across two trial periods with three experimental diets. Each trial period lasted eight days, including five days for diet adaptation and three days for digesta collection. Three diets were tested, with wheat and extruded wheat serving as the only AA source. To determine the basal endogenous losses of crude protein (CP) and AAs, a N-free diet was utilized. The detailed dietary compositions and nutritional contents pertinent to the AA digestibility test are presented in Table 2.

The experimental setup ensured that the supplementation levels of vitamins and minerals met or exceeded the nutrient requirements for nursery pigs, as recommended by the NRC (2012) [20]. Each pig was individually housed in environmentally controlled rooms, maintained at a temperature of 23 °C and 70% humidity. The pigs were kept in stainless steel metabolic cages (1.4 m × 0.7 m × 0.6 m) equipped with slatted floors, nipple drinkers, and feeding troughs. The cages featured adjustable barriers to accommodate the size of the pigs. Feed was administered at 4% of BW, divided into two meals served at 0800 and 1700 h each day, while water was provided ad libitum throughout the experiment. Sample collection adhered to protocols established by a previous study [18], ensuring consistency and reliability in data gathering.

Exp. 2 aimed to evaluate the effect of extruded wheat on growth performance, health status, nutrient digestibility, and fecal microbial composition of weaned piglets. According to the research conducted by Ma et al. [1], we determined that the proportion of extruded wheat incorporated in this study was to be set at 35%. A total of ninety-six healthy weaned piglets (Duroc × (Landrace × Large White), weaned at 28 days of age) with an initial BW of 8.58 ± 0.52 kg, were selected for the study. These piglets were divided into two groups (CON and 35% extruded wheat group) based on BW. Each group consisted of six pens, with 8 piglets (4 barrows and 4 gilts) per pen. To ensure uniform nutritional levels, diet formulations were adjusted, and vitamin and mineral supplementation was provided to meet or exceed the nutrient requirements for nursery pigs, as recommended by the NRC (2012) [20]. The piglets were housed in pens measuring 1.5 × 1.5 m^2^, equipped with plastic slatted floors, automatic stainless steel nipple drinkers, and feeders. The environmental conditions within the facility were consistently controlled and maintained by the farm to ensure optimal living conditions for the piglets. The ingredient compositions and nutrient levels of the experimental diets are shown in Table 3. Pigs were immunized in accordance with the established management protocols of the testing facility, aiming to prevent the outbreak of epidemic diseases.

The feeding trial was structured into two distinct phases: phase 1, spanning from d 0 to d 14, and phase 2, from d 15 to d 28. Throughout the experiment, individualBW of the piglets were recorded on d 0, d 15, and d 28, while feed consumption for each pen was documented for each period. From these data, the average daily gain (ADG), average daily feed intake (ADFI), and gain-to-feed ratio (G:F) were calculated per pen [21]. Diarrhea incidences in each piglet were monitored and recorded daily [22]. To assess digestibility, fecal samples were collected from pen units between d 25 and d 27. On d 27, blood samples were drawn from the anterior vena cava of pigs representing approximately average BW in each pen, using non-anticoagulant vacuum blood collection tubes (Greiner Bio-One GmbH, Frickenhausen, Austria). Finally, on d 28, twelve fresh fecal samples from each pen were collected via rectal palpation, immediately immersed in liquid nitrogen, and subsequently stored at −80 °C.

### 2.4. Chemical Analysis

Wheat, extruded wheat, all feed samples, and all fecal samples were ground using a 1 mm (40 mesh) sieve to ensure uniformity. The gross energy (GE) was determined with bomb calorimetry (Model 6400; Parr Instruments, Moline, IL, USA). The analysis of dry matter (DM), CP, ether extract (EE), and organic matter (OM) was conducted following the AOAC methods [23]. For the determination of neutral detergent fiber (NDF) and acid detergent fiber (ADF), filter bags (Model F57, Ankom Technology, Macedon, NY, USA) and a fiber analyzer (ANKOM200 Fiber Analyzer, Ankom Technology, Macedon, NY, USA) were utilized [24]. Additionally, the acid-insoluble ash (AIA) content in the feeds and feces from Exp. 2 was measured according to ISO methods [25]. This AIA content served as an endogenous marker for calculating the digestibility of the diets.

Concentrations of serum biochemical were assessed using a fully automatic biochemical analyzer (Model 7170, Hitachi, Tokyo, Japan). The concentrations of serum antioxidants, including superoxide dismutase (SOD), total antioxidant capacity (T-AOC), catalase (CAT), and glutathione peroxidase (GSH-Px), as well as inflammatory markers such as interleukin-6 (IL-6), interleukin-8 (IL-8), interleukin-10 (IL-10), and tumor necrosis factor-alpha (TNF-α), were measured using commercial ELISA kits. All ELISA kits were procured from Nanjing Jiancheng Bioengineering Institute (Nanjing, China), and the assays were performed in strict accordance with the protocols provided by the manufacturer. The ELISA conducted during the testing utilized the iMARK model (BIO-RAD, Hercules, CA, USA).

Total microbial genomic DNA was extracted from fecal samples using the QIAamp Fast DNA Stool Mini Kit (Qiagen, Hilden, Germany), following the manufacturer’s instructions. The quality and concentration of the extracted DNA were evaluated using 1.0% agarose gel electrophoresis and a NanoDrop2000 spectrophotometer (Thermo Scientific, Waltham, MA, USA). After assessment, the samples were stored at −80 °C for future use. The V3-V4 hypervariable regions of the bacterial 16S rRNA gene were then amplified on a T100 Thermal Cycler PCR (BIO-RAD, Hercules, CA, USA) using primers 338F (5′-ACTCCTACGGGAGGCAGCAG-3′) and 806R (5′-GGACTACHVGGGTWTCTAAT-3′). The optimized conditions for PCR amplification were as follows: an initial denaturation at 95 °C for 3 min, followed by 27 cycles of 95 °C for 30 s, 55 °C for 30 s, and 72 °C for 45 s, ending with a final extension at 72 °C for 10 min. The reaction (20 μL) included 4 μL of 5× FastPfu Buffer, 2 μL of 2.5 mM dNTPs, 0.8 μL each of 5 μM Forward and Reverse Primers, 0.4 μL of FastPfu Polymerase, 0.2 μL of BSA, 10 ng of Template DNA, and ddH2O to reach the final volume. The resulting PCR products were extracted from 2% agarose gels and purified with a PCR Purification Kit (ShanghaiYuhua Life Science and Technology Development Co. Ltd., Shanghai, China) in accordance with the manufacturer’s instructions. Finally, the purified PCR products were quantified using a Qubit 4.0 fluorometer (Thermo Fisher Scientific, Waltham, MA, USA).

### 2.5. Computational Methods

In Exp. 1, the DE, ME, and apparent total tract digestibility (ATTD) of GE, DM, OM, CP, NDF, and ADF were calculated using methods reported by a previous study [26]. Additionally, the apparent ileal digestibility (AID) and SID of CP and AA were determined using formulas established by Stein et al. [27]. In Exp. 2, the ATTD of nutrients was evaluated by measuring the concentration of AIA in both diets and feces, following the formulas described by Liu et al. [28].

### 2.6. Statistical Analysis

Outliers in the data were identified using the UNIVARIATE procedure in SAS 9.2 [29], and ANOVA analysis was conducted using the General Linear Model (GLM) method. For Exp. 1, each pig served as an experimental unit, with dietary treatment groups considered as fixed effects. In Exp. 2, each pen was considered as the experimental unit, and ANOVA analysis was performed on growth performance, diarrhea incidence, nutrient digestibility, and serum parameters, with diet as a fixed effect. The mean values for each treatment group were calculated using the LSMEANS statement, and multiple comparisons were conducted using the Tukey test. Values are presented as least squares means and standard error of the mean (SEM). Bioinformatics analysis of fecal microbiota was carried out using the Majorbio Cloud platform (https://cloud.majorbio.com, accessed on 10 December 2023). The alpha diversity of the samples was assessed using the Ace, Shannon, and Simpson indices. Similarity among microbial communities in different samples was evaluated through principal coordinate analysis (PCoA) based on Bray–Curtis dissimilarity. Differences in alpha diversity and differential microbial taxa between groups were identified using the Wilcoxon rank-sum test. Statistical significance was declared at *p* < 0.05 and tendency at 0.05 < *p* < 0.10.

## 3. Results

### 3.1. Effect of Extrusion on Available Energy Value and Nutrient Digestibility of Wheat

#### 3.1.1. Chemical Composition

As shown in Table 4, the extrusion increased GE, DM, and CP, but decreased NDF and ADF content of wheat. Conversely, there was a decrease in the levels of OM and EE. Additionally, the extrusion and expansion process significantly enhanced the concentration of the majority of AAs present in wheat.

#### 3.1.2. Energy Content and Nutrient Digestibility of Wheat and Extruded Wheat

Table 5 shows the available energy and the ATTD of nutrients in wheat and extruded wheat. The results indicate that the DE for piglets fed with wheat and extruded wheat were 15.16 and 16.08 MJ/kg DM, respectively, while the ME values were 14.59 and 15.66 MJ/kg DM, respectively. The extrusion process significantly increased the DE and ME of wheat (*p* < 0.05). Moreover, this processing method significantly enhanced the ATTD of GE, DM, OM, CP, and NDF (*p* < 0.05).

Table 6 and Table 7 show the effect of extrusion on the AID and SID of AAs in wheat. The results indicated that extrusion significantly enhanced the AID and SID of indispensable AAs in piglets (*p* < 0.05). Furthermore, for dispensable AAs, the extrusion notably improved the AID and SID of Ala, Asp, Glu, and Tyr (*p* < 0.05).

### 3.2. Effects of Extruded Wheat on Growth Performance, Diarrhea Rate, Nutrient Digestibility, and Serum Profiles of Weaned Piglets

#### 3.2.1. Growth Performance and Diarrhea Rate

Table 8 indicates that the inclusion of 35% extruded wheat at varying levels did not significantly affect the growth performance or the incidence of diarrhea in weaned piglets (*p* > 0.05).

#### 3.2.2. Nutrient Digestibility

Table 9 shows that dietary inclusion of 35% extruded wheat did not significantly influence the ATTD of nutrients in piglets (*p* > 0.05).

#### 3.2.3. Biochemical Parameters, Antioxidative Index and Cytokines in Serum

Table 10 shows the effect of dietary incorporation of 35% extruded wheat for weaned piglets, specifically examining serum biochemical parameters, antioxidative index, and cytokines. The analysis revealed that the inclusion of 35% extruded wheat did not significantly alter these parameters in weaned piglets (*p* > 0.05).

#### 3.2.4. Gut Microbial Composition

**Figure 1:**
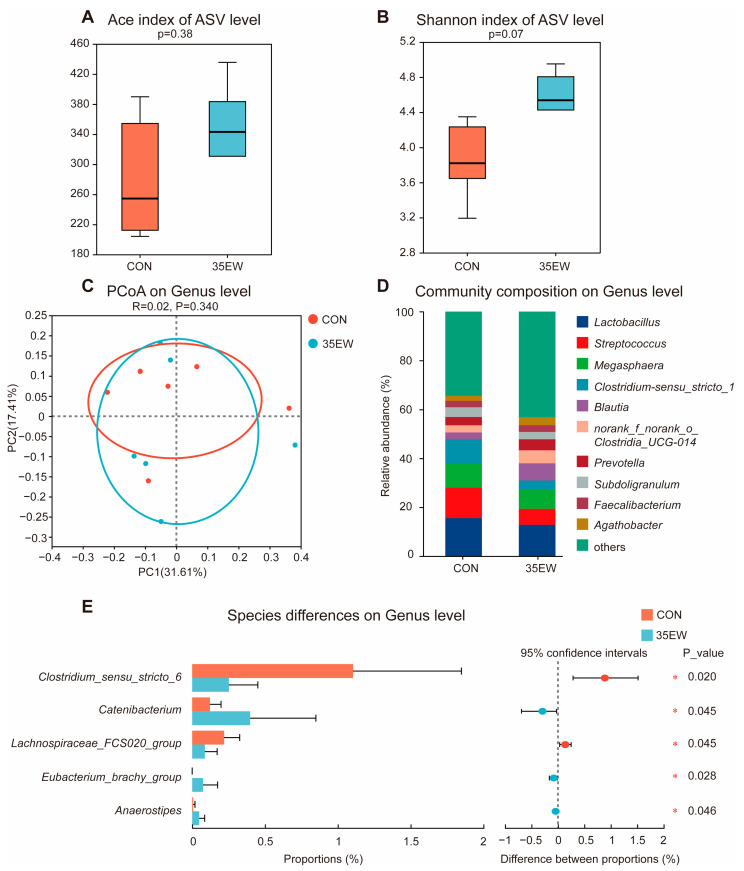
Dietary effect of 35% extruded wheat on fecal microbial composition and structure of weaned piglets: (**A**) Ace index; (**B**) Shannon index; (**C**) PCoA at genus level; (**D**) community composition at the genus level among the CON and 35EW groups; (**E**) differential bacteria at the genus level among the CON and 35EW groups. CON: corn–soybean meal-based diet; 35EW: diet supplemented with 35% extruded wheat. * represents the statistically significant differences among 2 groups, *p* < 0.05. *n* = 6.

The analysis of the microbial composition and structure in the fecal samples of weaned piglets from the CON (corn–soybean meal-based diet) and 35EW (diet supplemented with 35% extruded wheat) groups is shown in Figure 1. The Ace index showed no significant difference between the groups (Figure 1A), and there was a trend towards an increased Shannon index in 35EW (*p* = 0.07, Figure 1B). A PCoA based on Bray–Curtis dissimilarity indicated that the CON and 35EW groups clustered together, suggesting similar overall community structures (Figure 1C). Despite this clustering, community bar charts, which illustrated genus-level species composition for both CON and 35EW, showed no pronounced changes in community composition at the genus level between the two groups (Figure 1D). However, a comparative analysis of the microbiota between the two groups identified a significant difference in the relative abundance of *Clostridium_sensu_stricto_1*, *Catenibacterium, Eubacterium_brachy_group*, *Lachnospiraceae_FCS020_group*, and *Anaerostipes* (*p* < 0.05, Figure 1E).

## 4. Discussion

In the present study, the extrusion process significantly elevated the GE and CP levels in wheat, along with the concentrations of AAs. Such changes in nutrient content are commonly observed and are attributed to the reduction in moisture content that occurs during the extrusion process [30]. Specifically, exposure to high temperatures and pressures followed by rapid decompression leads to flash evaporation, thereby decreasing the water content in the material. The DE and ME values of both wheat and extruded wheat in this study were marginally lower than those reported in previous study [13], and this difference could potentially be ascribed to disparities in the origins of the raw materials or to the specific extrusion parameters employed. Nonetheless, the observed increase in DE and ME was closely linked to the increased digestibility of nutrients, which aligns with the finding of our earlier research [30]. Extrusion has been shown to enhance the solubility of nutrients, particularly dietary fibers, which are inherently difficult to digest, thus increasing their accessibility to digestive enzymes and improving digestibility [31]. Wheat is rich in NSP, which can hinder nutrient digestibility, often requiring the supplementation of specific enzyme preparations to counteract this effect [8]. In this study, we observed that the extrusion significantly increased the ATTD of various nutrients, including GE, CP, and NDF, highlighting the effectiveness of the extrusion process in enhancing nutrient digestibility.

Previous studies have shown that the available energy, as well as the AID and SID of AAs in wheat and corn, are nearly equivalent, indicating similar nutritional values between these two grains [4,32]. However, the digestion of other nutrients can be compromised due to the absence of endogenous enzymes required to break down NSPs, limiting the overall digestive efficiency of wheat and corn. Gao et al. [16] demonstrated that the extrusion converts α-helices into β-sheets in wheat germ protein, significantly improving its in vitro digestibility due to these structural changes. In our experiments, ileal cannulation tests revealed a significant increase in both AID and SID of indispensable AAs in pigs fed with extruded wheat. It has been proposed that extrusion has a dual impact on proteins, involving both depolymerization and modification effects [33]. During the process, depolymerization disrupts the macrostructure of proteins, which in turn may influence their sensitivity to enzymatic activity. In fact, these structural changes increase the accessibility of proteins to enzymes, thereby enhancing their digestibility. This enhancement of protein digestibility is a critical factor in how extrusion improves the nutritional value of feed. Overall, extrusion enhances the feed value of wheat, enabling its direct incorporation into pig diets.

In some countries, the economic and nutritional value of grains like wheat is often defined relative to corn [34]. Consequently, when corn prices rise, nutritionists look for alternatives to lower feed costs, with wheat being a promising substitute. However, research indicates that wheat-based diets, in comparison to corn-based diets, typically result in a reduced ADG in pigs [35]. The addition of NSP-degrading enzymes can alleviate this effect, allowing wheat to effectively replace corn as an energy source. The combined application of NSP enzymes with wheat not only improves ADG but also enhances G:F [36,37]. In the present experiment, pigs fed a diet containing 35% extruded wheat showed no significant difference in growth performance or incidence of diarrhea compared to those on corn-based diets. This finding suggests that the extrusion improves the feeding value of wheat, making it a viable alternative to corn in pig diets. The soluble NSPs present in wheat are known to increase the viscosity of gastric and intestinal digesta, which is likely a key factor contributing to the reduced feed utilization and growth performance observed in pigs consuming a wheat-based diet [38,39]. Extrusion degrades a portion of NSPs in wheat, effectively emulating the action of NSP enzymes, which is likely responsible for the consistent growth performance observed in the experiment.

Ensuring animal health and enhancing the growth performance are equally vital objectives. Serum biochemical markers serve as indicators of an animal’s health status and metabolic level, making them crucial for evaluating the efficacy of feed applications [40]. In this experiment, the inclusion of 35% extruded wheat in the diet of weaned piglets did not affect serum biochemical markers. This finding aligns with previous research, which suggests that substituting some corn with wheat in the diets of growing pigs does not significantly alter their blood antioxidative capacity [1]. Serum immunological and antioxidative markers, such as serum total protein (TP) and albumin (ALB), provide indirect insights into the health and metabolic state of the body [41]. The absence of differences in these markers suggests that the inclusion of 35% extruded wheat does not adversely affect the antioxidative capacity or overall health status of weaned piglets.

There is a well-established correlation between diet and the host microbiome, emphasizing the significant role of macronutrients in shaping gut health [42]. Carbohydrates, in particular, are pivotal in influencing the composition and functionality of the gut microbiome [43]. Zhang et al. [44] revealed that various sources of carbohydrates significantly impact the efficiency of xylanase supplementation and play a vital role in determining the diversity of the microbiome within piglet intestines. In the study, 35% extruded wheat in the diet of weaned piglets was found to increase fecal microbial diversity. This increase in diversity is significant because it may contribute to a healthier and more stable microbial ecosystem, which is crucial for promoting intestinal health. The NSPs present in wheat, such as arabinoxylans, β-glucans, and pectic polysaccharides, may be responsible for these changes [4]. These NSPs can exert prebiotic effects through their hydrolyzed products, potentially leading to alterations in the gut microbiome. Ma et al. [1] suggested that substituting corn with wheat in the diets of growing pigs enhances intestinal health by increasing the relative abundance of beneficial gut bacteria. This finding is consistent with the results of our experiment, which demonstrated that the inclusion of 35% extruded wheat significantly augmented the populations of *Lachnospiraceae_FCS020_group* and *Anaerostipes*. *Lachnospiraceae*, a family of bacteria identified as potentially beneficial, plays a crucial role in the metabolism of various carbohydrates by fermenting fibrous disaccharides and pectin, thereby producing acetate and butyrate, which serve as principal energy sources for the host [45]. Similarly, *Anaerostipes* is known for its ability to produce butyrate. Research indicated that *Anaerostipes caccae* CML199, through the butyrate-driven gut–bone axis, promotes bone development and positively influences skeletal health [46]. Thus, the dietary inclusion of wheat not only supports gut microbiota diversity, but also contributes to overall physiological benefits in growing pigs. Furthermore, the *Eubacterium_brachy_group* may be considered beneficial microbes, as *Eubacterium* is capable of producing butyrate, which plays a crucial role in reducing inflammation levels [47].

## 5. Conclusions

In summary, extrusion significantly increased the DE and ME, as well as the ATTD, of GE, CP, and NDF in wheat, while also improving both the AID and the SID of indispensable and most dispensable AAs in wheat. Notably, the inclusion of 35% extruded wheat in the diet does not affect the growth performance of weaned piglets, but increases the relative abundance of beneficial microbes in feces.

## Figures and Tables

**Table 1:** Composition and nutrient levels of the available energy experimental diets (%, as-fed basis).

Items	Wheat Diet	Extruded Wheat Diet
Wheat	96.80	—
Extruded wheat	—	96.80
Dicalcium phosphate	1.50	1.50
Limestone	0.90	0.90
NaCl	0.30	0.30
Premix ^1^	0.50	0.50
Total	100.00	100.00
Nutrient levels ^2^		
Gross energy, MJ/kg	15.95	16.79
Dry matter	93.81	98.11
Organic matter	96.39	95.79
Crude protein	13.37	13.48
Ether extract	1.01	1.18
Neutral detergent fiber	15.53	14.54
Acid detergent fiber	3.10	2.95
Ash	3.61	4.21

^1^ The premix offered the following per kilogram of feed: 12,000 IU of vitamin A in the form of vitamin A acetate; 2500 IU of vitamin D as vitamin D3; 30 IU of vitamin E as DL-α-tocopheryl acetate; 12 μg of vitamin B12; 3 mg of vitamin K as menadione sodium bisulfate; 15 mg of D-pantothenic acid in the form of calcium pantothenate; 40 mg of nicotinic acid; 400 mg of choline as choline chloride; 30 mg of manganese as manganese oxide; 90 mg of iron as iron sulfate; 10 mg of copper as copper sulfate; 0.35 mg of iodine as ethylenediamine dihydroiodide; and 0.3 mg of selenium as sodium selenite. ^2^ Measured value.

**Table 2:** Composition and nutrient levels of the amino acid digestibility experimental diets (%, as-fed basis).

Items	N-Free Diet	Wheat Diet	Extruded Wheat Diet
Corn starch	74.30	—	—
Wheat	—	96.60	—
Extruded wheat	—	—	96.60
Soybean oil	3.00	—	—
Cellulose acetate	4.00	—	—
Sucrose	15.00	—	—
Limestone	0.40	0.80	0.80
NaCl	0.30	0.30	0.30
Dicalcium phosphate	2.20	1.50	1.50
Chromic oxide	0.30	0.30	0.30
Premix ^1^	0.50	0.50	0.50
Total	100.00	100.00	100.00
Nutrient levels ^2^			
Crude protein	0.94	13.44	13.91
Indispensable amino acids			
Arg	0.01	0.55	0.55
His	0.01	0.31	0.29
Leu	0.02	0.43	0.41
Ile	0.06	0.78	0.77
Lys	0.01	0.33	0.35
Met	0.00	0.14	0.19
Phe	0.08	0.57	0.51
Thr	0.03	0.36	0.35
Trp	0.00	0.14	0.14
Val	0.04	0.55	0.54
Dispensable amino acids			
Ala	0.03	0.45	0.44
Asp	0.04	0.62	0.62
Cys	0.10	0.28	0.38
Glu	0.15	3.53	3.46
Gly	0.02	0.51	0.50
Pro	0.09	0.93	1.04
Ser	0.04	0.52	0.52
Tyr	0.04	0.28	0.27

^1^ The premix offered the following per kilogram of feed: 12,000 IU of vitamin A in the form of vitamin A acetate; 2500 IU of vitamin D as vitamin D3; 30 IU of vitamin E as DL-α-tocopheryl acetate; 12 μg of vitamin B12; 3 mg of vitamin K as menadione sodium bisulfate; 15 mg of D-pantothenic acid in the form of calcium pantothenate; 40 mg of nicotinic acid; 400 mg of choline as choline chloride; 30 mg of manganese as manganese oxide; 90 mg of iron as iron sulfate; 10 mg of copper as copper sulfate; 0.35 mg of iodine as ethylenediamine dihydroiodide; and 0.3 mg of selenium as sodium selenite. ^2^ Measured value.

**Table 3:** Composition and nutrient levels of experimental diets (%, as-fed basis).

Items	CON	35% Extruded Wheat Diet
Corn	61.72	32.39
Extruded wheat	—	35.00
Soybean meal	12.46	6.73
Extruded full-fat soybean	5.00	5.00
Fish meal	3.00	3.00
Whey powder	6.00	6.00
Soy protein concentrate	6.00	6.00
Soybean oil	2.20	2.20
Dicalcium phosphate	1.00	1.00
Limestone	0.80	0.82
NaCl	0.20	0.20
*L*-Lysine-HCl	0.54	0.63
*DL*-Methionine	0.10	0.11
*L*-Threonine	0.23	0.20
*L*-Tryptophan	0.06	0.05
*L*-Valine	0.03	0.03
Zinc oxide	0.16	0.16
Premix ^1^	0.50	0.50
Total	100.00	100.00
Nutrient levels ^2^		
ME, MJ/kg	17.03	17.09
Crude protein	18.30	18.23
SID Lys	1.45	1.45
SID Met	0.39	0.39
SID Thr	0.72	0.72
SID Trp	0.23	0.23
SID Val	0.81	0.81
Calcium	0.84	0.83
Available phosphorus	0.46	0.46

^1^ The premix offered the following per kilogram of feed: 12,000 IU of vitamin A in the form of vitamin A acetate; 2500 IU of vitamin D as vitamin D3; 30 IU of vitamin E as DL-α-tocopheryl acetate; 12 μg of vitamin B12; 3 mg of vitamin K as menadione sodium bisulfate; 15 mg of D-pantothenic acid in the form of calcium pantothenate; 40 mg of nicotinic acid; 400 mg of choline as choline chloride; 30 mg of manganese as manganese oxide; 90 mg of iron as iron sulfate; 10 mg of copper as copper sulfate; 0.35 mg of iodine as ethylenediamine dihydroiodide; and 0.3 mg of selenium as sodium selenite. ^2^ CP was a measured value, while the others were calculated values.

**Table 4:** Chemical component composition of wheat and extruded wheat (%, as-fed basis).

Items	Wheat	Extruded Wheat
Gross energy, MJ/kg	16.48	17.24
Dry matter	94.10	97.99
Organic matter	98.49	98.30
Crude protein	13.45	14.07
Ether extract	1.52	1.44
Neutral detergent fiber	16.34	15.21
Acid detergent fiber	2.53	2.45
Calcium	0.14	0.15
Phosphate	0.32	0.29
Indispensable amino acids		
Arg	0.57	0.58
His	0.29	0.30
Leu	0.80	0.82
Ile	0.41	0.43
Lys	0.37	0.34
Met	0.23	0.18
Phe	0.57	0.56
Thr	0.36	0.38
Trp	0.15	0.15
Val	0.56	0.55
Dispensable amino acids		
Ala	0.45	0.46
Asp	0.62	0.65
Cys	0.37	0.30
Glu	3.52	3.74
Gly	0.51	0.53
Pro	1.16	1.32
Ser	0.55	0.58
Tyr	0.29	0.29

**Table 5:** Effect of extrusion on the available energy and nutrient digestibility of wheat (%, DM basis).

Items	Wheat	Extruded Wheat	SEM	*p* Value
Energy content, MJ/kg				
DE	15.16	16.08	0.16	<0.01
ME	14.59	15.66	0.20	<0.01
ME/DE	96.21	97.40	0.52	0.28
ATTD, %				
GE	86.58	90.79	0.77	<0.01
DM	87.87	90.84	0.57	<0.01
OM	89.85	92.42	0.52	<0.01
CP	79.28	85.44	1.18	<0.01
NDF	61.39	74.36	2.14	<0.01
ADF	47.65	50.77	2.02	0.47

DE, digestible energy; ME, metabolizable energy; ATTD, apparent total tract digestibility; GE, gross energy; DM, dry matter; OM, organic matter; CP, crude protein; EE, ether extract; NDF, neutral detergent fiber; ADF, acid detergent fiber. *n* = 6.

**Table 6:** Effect of extrusion on the apparent ileal digestibility of crude protein and amino acids of wheat (%).

Items	Wheat	Extruded Wheat	SEM	*p* Value
CP	74.66	82.61	2.16	0.07
Indispensable amino acids				
Arg	79.10	84.99	1.33	0.02
His	80.66	87.79	1.48	<0.01
Ile	78.86	87.90	1.61	<0.01
Leu	81.20	87.14	1.17	<0.01
Lys	62.70	75.54	2.55	<0.01
Met	83.98	89.23	1.35	0.04
Phe	82.93	89.44	1.18	<0.01
Thr	66.54	76.04	2.27	0.03
Trp	71.80	84.47	2.76	0.01
Val	76.19	85.34	1.62	<0.01
Dispensable amino acids				
Ala	63.67	77.36	2.74	<0.01
Asp	67.26	76.59	2.16	0.02
Cys	81.38	82.05	1.47	0.83
Glu	91.36	94.72	0.64	<0.01
Gly	61.87	66.74	3.49	0.52
Pro	73.28	78.80	3.47	0.54
Ser	78.61	83.69	1.39	0.06
Tyr	77.72	83.94	1.26	<0.01

*n* = 6.

**Table 7:** Effect of extrusion on the standardized ileal digestibility of crude protein and amino acids of wheat ^1^ (%).

Items	Wheat	Extruded Wheat	SEM	*p* Value
CP	84.09	91.86	2.14	0.07
Indispensable amino acids				
Arg	85.29	91.44	1.36	0.01
His	86.48	93.26	1.44	<0.01
Ile	84.90	93.60	1.50	<0.01
Leu	87.13	93.13	1.17	<0.01
Lys	72.64	86.60	2.71	<0.01
Met	86.67	92.97	1.48	0.02
Phe	87.75	93.93	1.13	<0.01
Thr	77.50	87.13	2.28	0.02
Trp	81.09	93.69	2.75	0.01
Val	82.64	91.82	1.62	<0.01
Dispensable amino acids				
Ala	74.08	87.85	2.93	<0.01
Asp	76.48	86.14	2.19	0.02
Cys	88.19	91.62	1.56	0.30
Glu	93.64	97.02	0.64	<0.01
Gly	76.76	81.87	3.50	0.50
Pro	90.83	90.15	4.17	0.95
Ser	85.51	90.70	1.39	0.06
Tyr	84.55	90.74	1.26	<0.01

^1^ Values for SID were calculated by correcting the AID values with the basal endogenous losses. Basal endogenous losses (g/kg DM intake) averaged as CP, 13.40 Trp, 0.14; Cys, 0.28; Asp, 0.64; Arg, 0.38; His, 0.18; Gly, 0.83; Ile, 0.28; Leu, 0.51; Lys, 0.39; Met, 0.06; Phe, 0.27; Thr, 0.43; Ser, 0.40; Glu, 0.88; Pro, 2.02; Val, 0.39; Ala, 0.51; Tyr, 0.21. *n* = 6.

**Table 8:** Effect of dietary supplementation of 35% extruded wheat level on growth performance and diarrhea rate of weaned pigs.

Items	CON	35%Extruded Wheat Diet	SEM	*p* Value
Initial BW, kg	8.59	8.57	0.52	0.98
Medial BW, kg	13.25	13.15	0.77	0.96
Final BW, kg	18.63	18.43	0.90	0.92
Growth performance				
d 0~14				
ADG, g/d	332	328	20.53	0.92
ADFI, g/d	576	584	16.89	0.82
G:F	1.75	1.78	0.06	0.80
d 15~28				
ADG, g/d	385	377	12.03	0.76
ADFI, g/d	701	696	14.40	0.87
G:F	1.78	1.79	0.03	0.59
d 0~28				
ADG, g/d	358	352	14.79	0.85
ADFI, g/d	639	640	14.50	0.96
G:F	1.72	1.77	0.03	0.37
Diarrhea rate, %				
d 0~14	2.20	2.33	0.45	0.89
d 15~28	2.60	2.74	0.48	0.89
d 0~28	2.40	2.54	0.36	0.86

BW, body weight; ADG, average daily gain; ADFI, average daily feed intake; G:F, gain-to-feed ratio.

**Table 9:** Effect of dietary supplementation of 35% extruded wheat on nutrient digestibility in weaned pigs (%).

Items	CON	35% Extruded Wheat Diet	SEM	*p* Value
GE	78.63	77.65	0.81	0.57
DM	80.23	78.53	0.74	0.27
OM	83.40	81.87	0.65	0.26
CP	69.00	65.89	1.24	0.23
NDF	59.95	55.75	1.98	0.31
ADF	44.84	40.07	2.88	0.45

DE, digestible energy; ME, metabolizable energy; GE, gross energy; DM, dry matter; OM, organic matter; CP, crude protein; EE, ether extract; NDF, neutral detergent fiber; ADF, acid detergent fiber. *n* = 6.

**Table 10:** Effects of dietary supplementation of 35% extruded wheat on serum biochemical parameters, antioxidant activity, and inflammatory indices in weaned pigs.

Items	CON	35% Extruded Wheat Diet	SEM	*p* Value
Biochemical parameters				
TP, g/L	39.97	37.42	2.20	0.59
ALB, g/L	16.91	18.24	0.77	0.42
TC, mmol/L	1.85	2.06	0.14	0.48
TG, mmol/L	0.70	0.57	0.06	0.31
Antioxidant status				
SOD, U/mL	75.69	69.13	5.24	0.56
GSH-Px, U/mL	437.36	432.41	16.28	0.89
T-AOC, U/mL	10.66	10.71	0.44	0.96
MDA, nmol/mL	5.16	5.50	0.34	0.64
Inflammatory parameters				
IL-6, pg/mL	161.19	180.44	20.71	0.67
IL-8, pg/mL	82.06	72.96	3.70	0.54
IL-10, pg/mL	14.83	16.20	6.21	0.33
TNF-α, pg/mL	57.65	62.74	0.67	0.52

TP, total protein; ALB, albumin; TC, total cholesterol; TG, triglyceride; SOD, superoxide dismutase; GSH-Px, glutathione peroxidase; T-AOC, total antioxidant capacity; MDA, malondialdehyde; IL-6, interleukin-6; IL-8, interleukin-8; IL-10, interleukin-10; TNF-α, tumor necrosis factor-α. *n* = 6.

## Data Availability

The original contributions generated for this study are included in the article; further inquiries can be directed to the corresponding author.
